# Mesoporous Cobalt Ferrite Nanosystems Obtained by Surfactant-Assisted Hydrothermal Method: Tuning Morpho-structural and Magnetic Properties via pH-Variation

**DOI:** 10.3390/nano10030476

**Published:** 2020-03-06

**Authors:** Petru Palade, Cezar Comanescu, Andrei Kuncser, Daniela Berger, Cristian Matei, Nicusor Iacob, Victor Kuncser

**Affiliations:** 1 National Institute of Materials Physics, Atomistilor 405A, 077125 Magurele, Romania; palade@infim.ro (P.P.); akuncser@yahoo.com (A.K.); nicusor.iacob@infim.ro (N.I.); kuncser@infim.ro (V.K.); 2 Polytechnic University of Bucharest, Faculty of Applied Chemistry and Materials Science 1–7 Polizu St, 011061 Bucharest, Romania; danaberger01@yahoo.com (D.B.); cristi_matei@yahoo.com (C.M.)

**Keywords:** mesoporous cobalt ferrite, magnetic nanoparticles, Mössbauer spectroscopy, magnetic properties adjusted via pH variation

## Abstract

A facile and cheap surfactant-assisted hydrothermal method was used to prepare mesoporous cobalt ferrite nanosystems with BET surface area up to 151 m^2^/g. These mesostructures with high BET surface areas and pore sizes are made from assemblies of nanoparticles (NPs) with average sizes between 7.8 and 9.6 nm depending on the initial pH conditions. The pH proved to be the key factor for controlling not only NP size, but also the phase purity and the porosity properties of the mesostructures. At pH values lower than 7, a parasite hematite phase begins to form. The sample obtained at pH = 7.3 has magnetization at saturation M_s_ = 38 emu/g at 300 K (54.3 emu/g at 10 K) and BET surface area S_BET_ = 151 m^2^/g, whereas the one obtained at pH = 8.3 has M_s_ = 68 emu/g at 300 K (83.6 emu/g at 10 K) and S_BET_ = 101 m^2^/g. The magnetic coercive field values at 10 K are high at up to 12,780 Oe, with a maximum coercive field reached for the sample obtained at pH = 8.3. Decreased magnetic performances are obtained at pH values higher than 9. The iron occupancies of the tetrahedral and octahedral sites belonging to the cobalt ferrite spinel structure were extracted through decomposition of the Mössbauer patterns in spectral components. The magnetic anisotropy constants of the investigated NPs were estimated from the temperature dependence of the hyperfine magnetic field. Taking into consideration the high values of BET surface area and the magnetic anisotropy constants as well as the significant magnetizations for saturation at ambient temperature, and the fact that all parameters can be adjusted through the initial pH conditions, these materials are very promising as recyclable anti-polluting agents, magnetically separable catalysts, and targeted drug delivery vehicles.

## 1. Introduction 

The applicative potential of ferrite nanoparticles (NPs) is enormous in various technological fields such as permanent magnets [1], biomedicine [2], catalysis [3], electromagnetic shielding [4], electronic devices [5], magnetic nanofluids (heat transfer [6], magnetorheology [7]), anti-pollution agents [8], building industry [9], electrochemical energy storage [10], and biosensors [11].

Magnetic nanostructures have aroused keen interest in the scientific community due to their different physical and chemical properties when compared to the corresponding bulk materials. An important candidate among the ferrite-type materials which is useful for various applications is cobalt ferrite (CoFe_2_O_4_). In the ideal case, it has an inverse spinel structure with Fe^3+^ ions occupying both tetrahedral A positions and octahedral B positions in the AB_2_O_4_ formula unit and Co^2+^ occupying the remaining octahedral B position. The magnetic moment of the ions belonging to the octahedral positions is oppositely oriented towards one of the ions from the tetrahedral positions, leading usually to a total uncompensated magnetic moment per formula unit. The magnetic anisotropy constant of CoFe_2_O_4_ is much higher than that of magnetite (Fe_3_O_4_) due to the magnetocrystalline contribution, providing semi-hard magnetic properties to CoFe_2_O_4_. Besides this, other properties such as significant magnetization at saturation, high coercive field and Curie temperature, and good chemical and magnetic stability make this material very interesting for a wide range of applications. Besides the excellent absorber properties in the microwave range, CoFe_2_O_4_ has a wide energy bandgap, with semiconductor behavior, providing electrocatalytic effect, making it suitable for energy harvesting and conversion. 

Various preparation methods have been used in order to obtain cobalt ferrite NPs involving co-precipitation [12], thermal decomposition of metal salts in solvents with high boiling temperature [13], sol–gel [14], microemulsions [15], polyols [16], hydrothermal methods [17], combustion [18], sonochemistry [19], and electrochemical methods [20]. Particularly, mesoporous cobalt ferrite structures are very promising as recyclable anti-polluting agents [21], magnetically separable catalysts [22], and targeted drug delivery vehicles [23]. For these applications, mesoporous CoFe_2_O_4_ particles must have high enough magnetization at saturation at ambient temperature (M_s_ more than 30 emu/g) and significant BET surface area. Mesoporous CoFe_2_O_4_ spherical structures with M_s_ = 60.2 emu/g and surface area S_BET_ of 85.4 m^2^/g were obtained by hydrothermal reaction using polyethylene glycol as surfactant and sodium acetate as electrostatic stabilizer [24]. Mesoporous CoFe_2_O_4_ particles, useful as catalysts in methane combustion reactions, were obtained by thermolysis of two new cobalt ferrioxalate coordination compounds [25] and have M_s_ = 36.3 emu/g and S_BET_ = 77.6 m^2^/g, and M_s_ = 23.6 emu/g and S_BET_ = 55.7 m^2^/g. Cobalt ferrite mesostructures built from nanocrystals, with M_s_ = 34.73 emu/g and S_BET_ = 60.4 m^2^/g, were very recently obtained by MOF-templated synthesis [21] and proven to be useful for degradation of bisphenol A. Recently, using a cationic surfactant (CTAB)-assisted solvothermal method [26], hollow mesoporous CoFe_2_O_4_ magnetic particles with M_s_ = 42.2 emu/g and S_BET_ = 58.03 m^2^/g were synthesized and shown to be suitable for a microwave-triggered controllable drug delivery system. All M_s_ values reported above were measured at ambient temperature.

In the present work, we describe a cheap and facile surfactant-assisted hydrothermal method to prepare mesoporous CoFe_2_O_4_ structures with high BET surface area and significant magnetization at saturation at ambient temperature starting from non-ionic block copolymer Pluronic™ P123 environmentally friendly surfactant. The synthetic method is environmentally benign since there are no toxic precursors involved (as is the case for thermal decomposition or microemulsions methods) and the experimental steps are straightforward. The mesoporous structures were made of NPs, as demonstrated by structural and morphological data. The sample obtained at pH = 7.3 has M_s_ = 38 emu/g and S_BET_ = 151 m^2^/g, whereas the one obtained at pH = 8.3 has M_s_ = 68 emu/g and S_BET_ = 101 m^2^/g, with both magnetization values being measured at ambient temperature. A complex magnetic and Mössbauer analysis was carried out in order to understand the magnetic behavior of the NPs under study in correlation with the local structure and electronic phenomena. These results were further corroborated with morphological, structural and compositional information extracted from X-ray diffraction, TEM, and BET measurements. 

## 2. Materials and Methods

In a typical synthesis, 4 g of a non-ionic block copolymer Pluronic™ P123 was mixed in aqueous acidic media, and 1 mmol Co(NO_3_)_2_.6H_2_O and 2 mmol Fe(NO_3_)_3_.9H_2_O were added under vigorous stirring. All reagents were purchased from Alfa Aesar, Kandel, Germany. The reaction pH was adjusted in the range 4–11 by titration of the initial acidic solutions with 10 M NaOH until the desired value of pH was reached for the solutions in the reaction vessel. The morphology and phase composition were found to be highly dependent on this parameter. The Teflon-lined vessel was then closed, and the temperature was raised up to 100 °C in 30 min, then solutions were thermally treated at 100 °C for 60 h, a period long enough for complete hydrothermal reaction to occur. The reaction gel was then centrifuged several times until neutral pH. The resulting brown clay was heated up to 550 °C with a ramp of 3 °C/min in a crucible and maintained at 550 °C for 2 h to obtain crystal lattice strengthening, followed by natural cooling down. The cobalt ferrite actually also exhibits an autocatalytic effect on the decomposition of the remaining surfactant, as the drying alone at 55 °C yields almost complete conversion to CoFe_2_O_4_. Further lattice strengthening at 550 °C would yield the final cobalt ferrite. The phase composition was further investigated by X-ray diffraction using a D8 Advance diffractometer with Cu K_α_ radiation (Bruker, Karlsruhe, Germany). FTIR measurements have been performed using a Spectrum BX (II) spectrometer (Perkin Elmer, Waltham, MA, USA) and for TGA-DSC investigations using a SetSys Evolution apparatus (Setaram, Caluire, France). The data for DSC have been collected in synthetic air (20% O_2_, 80% N_2_) using a heating rate of 5 °C/min and a gas flow of 16 mL/min. The distribution of the pores in the samples was extracted from nitrogen sorption isotherms measured with a Autosorb iQ_2_ gas sorption apparatus (Quantachrome, Boynton Beach, FL, USA), using BET method, N_2_, 77 K. The morphology of the obtained nanoparticles was studied by transmission electron microscopy (TEM) with a JEM-2100 electronic microscope (JEOL, Tokyo, Japan) equipped with tomography pole-piece, X-ray spectrometer, and ASTAR precession system for reciprocal space analysis. Magnetic properties of the samples were investigated at various temperatures in the range 10–300 K using a vibrating-sample magnetometer - VSM (Cryogenic Ltd., London, UK). ^57^Fe Mössbauer spectroscopy in transmission geometry allowed us to obtain more in-depth information concerning the magnetic and local structure of the nanoparticulate systems under study. For this purpose, we have used an integrated system built from a spectrometer (SEE Co., Minneapolis, MN, USA) operating under constant acceleration mode, a closed-cycle helium cryostat in which sample was installed and a ^57^Co(Rh) radioactive source. The Mössbauer spectra measured at temperatures ranging from 10 K up to ambient temperature were analyzed via the NORMOS program which allows the decomposition of the measured pattern in spectral components corresponding to different Fe non-equivalent positions.

## 3. Results and Discussion

### 3.1. X-ray Diffraction Analysis

**Figure 1:**
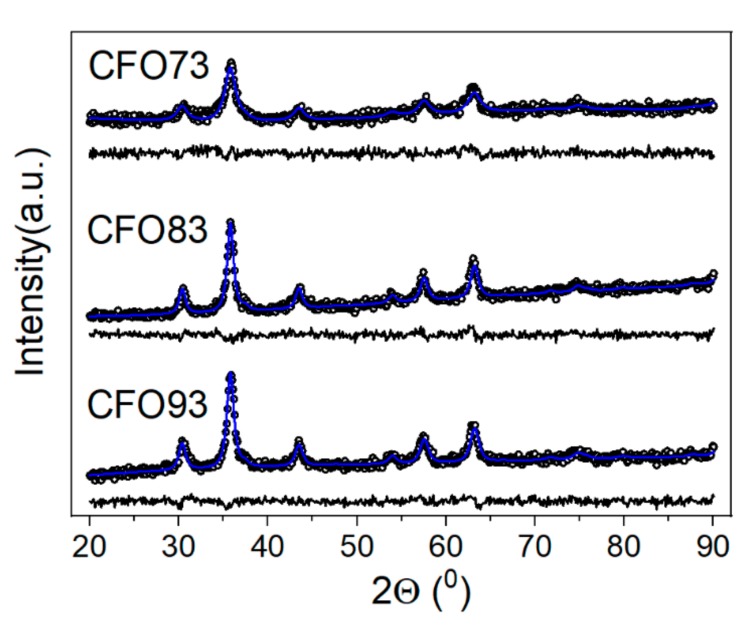
Rietveld refinements of XRD data for CFO73 (pH = 7.3), CFO83 (pH = 8.3), and CFO93 (pH = 9.3).

The morphology and composition of the samples were modified by changing the pH value. Only at pH values between 7 and 9.5 were the obtained samples pure and with well-crystallized phase according to diffraction data (Table 1, Figure 1, Supplementary Information S1).

One example is the sample obtained at pH 6.4, which was demonstrated to be a mixture of hematite (α-Fe_2_O_3_) and cobalt ferrite (CoFe_2_O_4_). The critical parameter to yield high-quality cobalt ferrite turned out to be the pH of the reaction mixture. Hence, pH values lower than 7 favor formation of a mixture of phases as confirmed by powder XRD (α-Fe_2_O_3_—hematite and CoFe_2_O_4_—cobalt ferrite), whereas at pH values higher than 7, cobalt ferrite is obtained as pure phase. It seems that basic conditions favor the formation of cobalt ferrite. Moreover, at pH values higher than 10, the samples have low crystallinity as observed from XRD data from Supplementary Information S1. Only the samples obtained at pH values of 7.3, 8.3, and 9.3 were hereafter selected, being denoted in the text as CFO73, CFO83, and CFO93, respectively. Rietveld refinements of XRD data are shown in Figure 1.

The software used for this purpose was MAUD [27]. For the fitting, we used the lognormal distribution of crystallite size allowed by MAUD software. The average value of the crystallite size for investigated samples is given in Table 2. Regarding the possible co-existence of Co_3_O_4_ and CoFe_2_O_4_ phases in the investigated samples reported herein, the authors rule this out on the following basis: the lattice parameter of bulk Co_3_O_4_ is 0.8083 nm (PDF file 04-005-4386, cubic crystallographic group Fd-3m), whereas the lattice parameter of bulk CoFe_2_O_4_ is 0.8377 nm (PDF file 04-006-4147, cubic crystallographic group Fd-3 m). The lattice parameter of our samples resulted from Rietveld refinements is between 0.8369 and 0.8371 nm (Table 2), very close to bulk CoFe_2_O_4_ value. There is a significant difference between the lattice parameter of bulk CoFe_2_O_4_ and bulk Co_3_O_4_ and no additional XRD peak assignable to Co_3_O_4_ in X-ray diffractograms of our samples can be detected. Besides the desired phase CoFe_2_O_4_, no parasite phases can be observed in the XRD data, in good agreement with Mössbauer spectra that have demonstrated the absence of wüstite, hematite, or other iron oxides. 

Also in the same table are the lattice constant and rms microstrain resulted from Rietveld refinements together with the reliability fit parameters. The lattice constant of cobalt ferrite nanoparticles in CFO73, CFO83, and CFO93 samples is basically the same within the error bar, and is close to 0.8377 nm (PDF file 04-006-4147) obtained for bulk CoFe_2_O_4_ (cubic crystallographic group Fd-3m). The rms microstrain value is also comparable for all samples. 

According to Table 2, the smallest crystallite size was obtained for CFO73, synthesized at the lowest pH value among all three samples. The same conclusion was obtained from the TEM micrographs and the smallest M_s_ value was also measured for the same sample, as discussed further on. This magnetic behavior can be easily justified by the fact that the contribution of surface (with disordered magnetic structure) relative to bulk counterpart is more significant in small crystallites.

### 3.2. TGA-DSC Analysis

The TGA-DSC analysis (Supplementary Information S2) shows that decomposition of the P123 Pluronic polymer (PEO_20_PPO_70_PEO_20_. MW = 5800 g/mol) occur in stages, and up to about 400 °C, the decomposition of surfactant can be considered almost complete (> 90 wt% loss). The transformation comprises in a sharp endothermic peak, due to phase transformation (solid–liquid phase), followed by dehydration and selective breakage of the PEO and PPO chains to eventually burn off and give CO_2_ and H_2_O. Given that surfactant decomposition occurs up to 410–420 °C, the calcination of the gel was decided at 550 °C, and was further tuned according to the manuscript data.

### 3.3. FTIR Comparative Analysis of Pluronic P123 and CFO73, CFO83, and CFO93

A complementary technique (FTIR spectroscopy) was employed to prove that after calcination at 550 °C, no trace of surfactant exists. The FTIR data show a faint shift at ~456 cm^−1^ corresponding to the ferrite metal stretching at octahedral (Oh) site—typically occupied by Co^2+^ and Fe^3+^ cations, and a broad one at ~590 cm^−1^ associated with the intrinsic stretching vibrations of the metal at the tetrahedral (Td) site of the ferrite structure, where Fe^3+^ resides. Other strong absorption peaks can be observed at 3400 cm^−1^ (wide absorption band, valence vibrations of metal-hydroxyl groups, Fe-OH and Co-OH), at 1622 cm^−1^ (interlayer water: oscillations of the H-O-H bond) and 1000 cm^−1^ (stretching vibrations of the Fe-O-H) (Supplementary Information S3). None of the three ferrite samples (CFO73, CFO83, and CFO93) present any peaks associated to the Pluronic P123 surfactant. In fact, no peaks corresponding to any C-H stretching or bending can be detected. For reference, a full FTIR assignment for pure P123 [28] is given as follows: 963 cm^−1^, CH_2_ antisymmetric rock; 1061 cm^−1^ (shoulder), C-O-C antisymmetric stretch, CH_2_ symmetric rock; 1103 cm^−1^, C-O-C symmetric stretch; 1150 cm^−1^ (shoulder), C-C stretching, C-O-C antisymmetric stretch; 1237 cm^−1^ and 1281 cm^−1^, CH_2_ antisymmetric–symmetric twist; 1342 cm^−1^, CH_2_ antisymmetric wag; 1373 cm^−1^, methyl group symmetric deformation; 2970 cm^−1^, C-H stretching of methyl group [28]. It is obvious that no peaks for the surfactant could be observed in our FTIR spectra of CFO samples, consistent with decomposition of the poloxamer P123 below 400 °C (calcination was performed under optimized conditions at 550 °C).

### 3.4. Porosimetry Measurements

**Figure 2:**
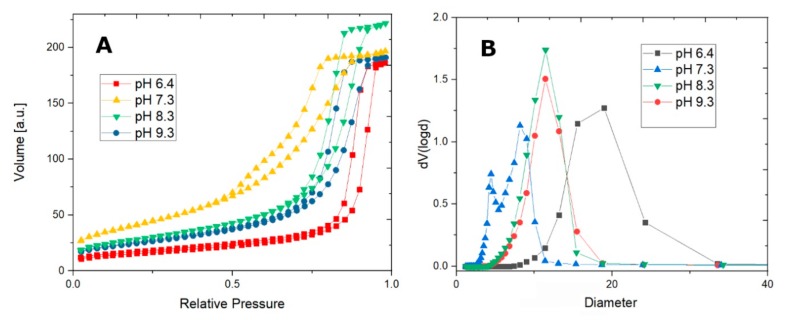
N_2_ sorption isotherms (**A**) and pore size distribution plot (**B**) for the cobalt ferrites synthesized pH values between 6.4 and 9.3.

N_2_ sorption isotherms show good morphological properties, as the samples are indeed mesoporous, with high pore volume (up to 0.34 cm^3^/g), high surface area (up to 151 cm^3^/g), and tunable pore sizes (5–11 nm) (Figure 2A). The pore size distribution is shown in Figure 2B. The BET surface area, pore volumes, and pore size diameters for CFO 73, CFO83, CFO93, and also for CoFe_2_O_4_@pH = 6.4 are shown in Table 1. The median pore sizes from our soft-nanocasting method are larger than the ones obtained for mesoporous cobalt ferrite from hard-nanocasting syntheses (when sacrificial mesoporous silica template was used, increasing the complexity and the synthesis time and cost). 

Samples synthesized at pH values between 6.4 and 9.3 show similar N_2_ sorption isotherms, exhibiting a type IV isotherm (according to IUPAC classification [29]), however, with slightly different hysteresis loops. Sample CoFe_2_O_4_@pH = 6.4 shows a type IVa (type IV hysteresis, type H1 hysteresis loop), whereas samples CFO73, CFO83, and CFO93 exhibit a type IVb hysteresis with an H2 hysteresis loop broader than at pH = 6.4 and with steeper desorption branch relative to the adsorption branch. Sample CoFe_2_O_4_@pH = 6.4, being chemically a hybrid phase hematite–cobalt ferrite, shows almost vertical parallel adsorption and desorption branches, with a slightly larger pore size of 15.50 nm but the BET surface area is the smallest among all samples (only 56 m^2^/g).

A phase composition change occurs at a slightly basic pH (pH = 7.3) as mentioned above. The PSD pore size distribution assessment shows two types of pore sizes arising from the bimodal desorption branch of the isotherm, and the hematite is now avoided as a side product at higher pH values (pH ≥ 7.3). BET analysis for CFO73 sample give the maximum surface area (151 m^2^/g) and the minimum pore size diameter (5.42 nm) among all studied samples. On the other hand, one may observe similar values of BET surface area and pore size for both CFO83 and CFO93 samples. Carrying out the synthesis at even higher pH does not change the pore size (11.4 nm on average), but the highest point of the isotherm decreases, accounting for a decreasing pore volume with pH increase. CFO83 and CFO93 samples are almost monodisperse, a fact confirmed by PSD analysis.

### 3.5. Transmission Electron Microscopy Analysis

**Figure 3:**
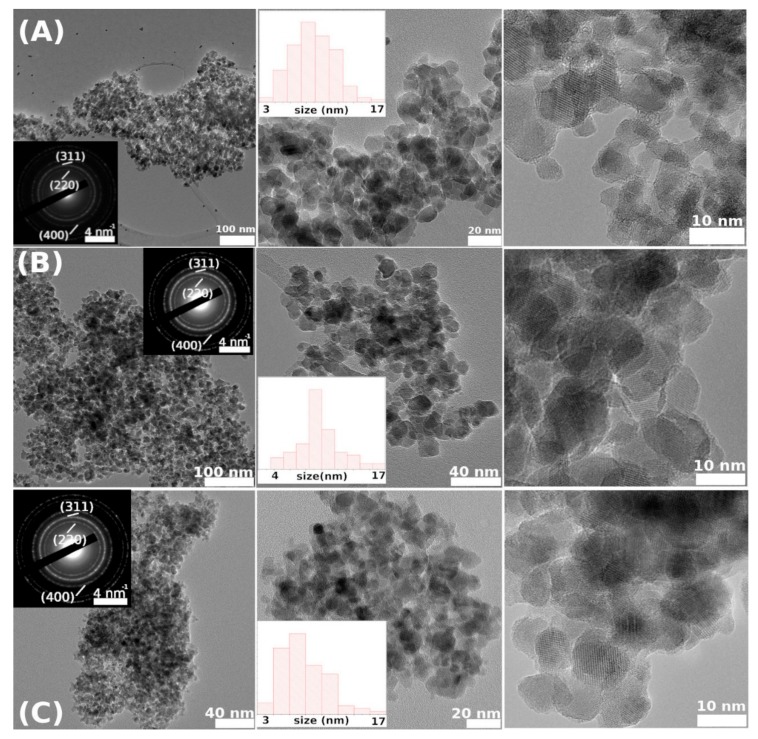
TEM and HRTEM micrographs for samples CFO73 (**A**), CFO83 (**B**), and CFO93 (**C**).

Analysis by conventional TEM imaging, HRTEM (high-resolution TEM), and selected area electron diffraction (SAED), and nanoparticle size histograms has been performed for each of the CFO73, CFO83, and CFO93 samples as shown in Figure 3 in rows (A), (B), and (C), respectively. The measurements show almost monodisperse CoFe_2_O_4_ nanoparticles (space group 227). Although the morphology appears similar, a closer look at the particle distribution provided noticeable differences (i.e., an average of 9 nm for CFO93, 9.6 nm for CFO83, and 7.8 nm for CFO73). EDAX (energy-dispersive absorption X-ray) measurements have been performed to estimate the stoichiometry of the samples. It has been demonstrated that the atomic ratio between Fe and Co is close to the theoretical value of 2 if we take into account the error bar of the method: 2.0(2) for sample CFO83 and 2.3(2) for sample CFO93. For sample CFO73, the results show a Fe to Co ratio higher than the theoretical one, with a negative influence on the magnetic properties, as subsequently described. 

### 3.6. Magnetic Measurements

**Figure 4:**
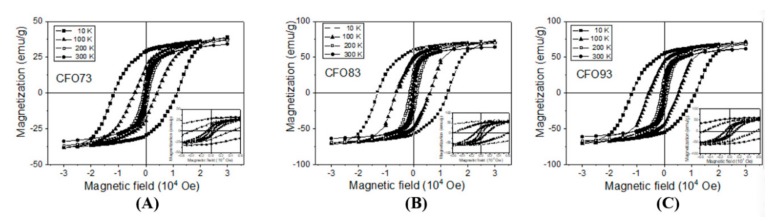
Hysteresis loops at selected temperatures in the range 10–300 K for samples CFO73 (**A**), CFO83 (**B**), and CFO93 (**C**).

Hysteresis loops measured at temperatures ranging from 10 to 300 K are shown for few representative temperatures in Figure 4A–C for samples CFO73, CFO83, and CFO93, respectively. One can see that saturation is achieved at magnetic fields above 30,000 Oe at 10 K, and at much lower magnetic fields at 300 K. Coercive magnetic fields (H_c0_) obtained at 10 K were 11,500, 12,780, and 11,610 Oe for samples CFO73, CFO83, and CFO93, respectively. These values can be correlated with the crystallite size; namely for CFO73, we obtained the smallest crystallite size from XRD and TEM data and the smallest value of the coercive field among all three samples, whereas for CFO83, the largest value for both the crystallite size and the coercive field among all samples was obtained. At the highest measurement temperature (300 K), the coercive field values were 250, 359, and 270 Oe for samples CFO73, CFO83, and CFO93, respectively. Also at this temperature, the largest coercive field was obtained for the sample with maximum crystallite size, and the lowest one for the sample with minimum crystallite size. The surface to volume ratio is the highest for the nanoparticles (NPs) with the lowest crystallite size which are contained in CFO73. Accordingly, the outer layer with distorted spin structure has the most significant magnetic effect for this sample. From the same hysteresis curves from Figure 4A–C, one can see that the lowest M_s_ value among all samples was found for CFO73 both at 10 and 300 K. One should recall that crystallites of cobalt ferrite with dimensions in the range obtained in this work (7–10 nm) which represent the building blocks of the mesoporous structures of CFO73, CFO83, and CFO93 should be single magnetic domain with Stoner–Wohlfarth-like behavior [30]. In such a nanoparticulate system of assumed non-interacting single magnetic domain NPs with the easy magnetic axis randomly distributed, the low temperature coercive magnetic field is H_c0_ = K/M_s_ for NPs with uniaxial anisotropy [30] and H_c0_ = 0.64 K/M_s_ for NPs with cubic anisotropy [31], where K is the magnetic anisotropy constant and M_s_ is the magnetization at saturation. Taking into account that H_c0_ has almost similar values at 10 K for all samples and that M_s_ is significantly smaller for CFO73 compared with CFO83 and CFO93, this sample must have the highest value of the anisotropy constant among all three samples. Another important aspect is that coercive field values measured at 300 K are small, but different from zero, and as a consequence, at least a fraction of NPs is not superparamagnetically relaxed, even at this temperature. The evolution of coercive field vs. temperature for non-interacting NPs is well described by the Kneller formula [32]:

(1) $$\;H_{c}=H_{c0}\left(1-{\left(\frac{T}{T_{B}}\right)}^{\frac{1}{2}}\right).$$

**Figure 5:**
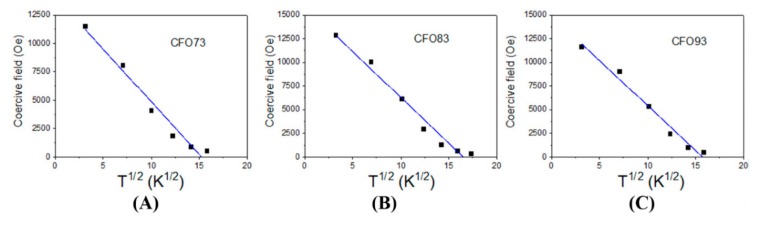
Dependence of coercive magnetic field on T^1/2^ for CFO73 (**A**), CFO83 (**B**), and CFO93 (**C**).

**Figure 6:**
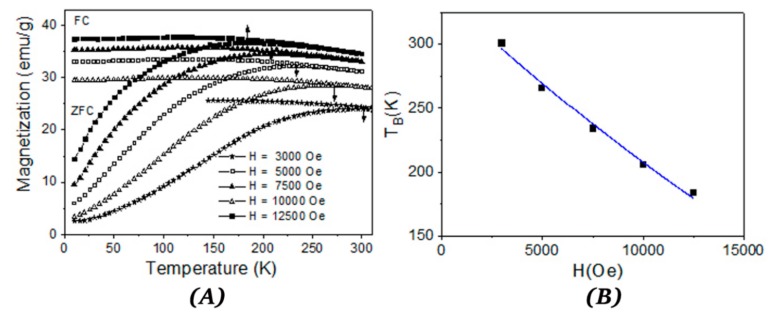
Zero field cooled/field cooled (ZFC-FC) curves at various fields (**A**) and blocking temperature (TB) dependence on applied magnetic field (**B**) for sample CFO73.

In the above formula H_c0_ is the coercive field extrapolated to 0 K, and T_B_ is the blocking temperature whereas T is the measuring temperature. For non-interacting NPs, this equation characterizes the transition from the magnetic frozen state to superparamagnetic relaxed state when the coercive magnetic field becomes zero. For CFO73, CFO83, and CFO93, the coercive field dependence on T^1/2^ is given in Figure 5A–C, respectively. The evolution of the experimental points was fitted with Kneller formula (Equation (1)). From the intersection between the fitted line and the x-axis (H_c_ = 0), we obtained the blocking temperatures (T_B_) of 233 K for CFO73, 273 K for CFO83, and 248 K for CFO93. As expected, the lowest blocking temperature was obtained for the sample with the smallest crystallite size (CFO73) and the largest blocking temperature was obtained for the sample with the largest crystallite size (CFO83). In Figure 5A–C one can see that the experimental points deviate from the fitted line at temperatures approaching the blocking temperatures. This suggests a distribution of particle size that means the existence of a fraction of NPs with dimensions larger than the mean value of the crystallite size which is not superparamagnetically relaxed at the blocking temperature obtained from the fit. Another explanation could be the microstructure of the samples obtained in the present work, which resembles a nanoporous foam with closed and open pores and significant surface area. The walls of this nanoporous foam are built from NPs with single magnetic domain behavior and magnetic dipolar interactions appear between these nanometer crystallites. Consequently, deviations from the model of non-interacting NPs assumed for derivation of Kneller formula could appear, leading to some uncertainties in the estimation of the blocking temperature (moreover, the time window of the measuring method providing the hysteresis loop is not defined). The way to find the blocking temperature (T_B_) more precisely is to plot the ZFC-FC (zero field cooled/field cooled) curves. The ZFC procedure assumes that the sample which is at high temperature in a superparamagnetic state is cooled down to the lowest measuring temperature without applying a magnetic field. Afterwards, a small magnetic field is applied in order to measure the magnetization when the temperature varies from the minimum to the maximum value, thus collecting the ZFC curve. At a certain temperature, NPs have enough thermal energy to overpass the magnetic energy barrier which separates the two possible magnetic orientations of the NPs (with the nanoparticle magnetic moment in the same direction and in the opposite direction to the applied magnetic field). Consequently, the NP magnetic moments align more on the direction of the magnetic field before providing a decreased time average value at even higher temperatures. The magnetization of the ZFC curve has a maximum at a specific temperature called the blocking temperature (T_B_). As the temperature continues to increase, the difference between the probabilities to orientate the particle along and opposite to the applied magnetic field decreases, and the total magnetization diminishes. The ZFC curve for CFO73 increases continuously up to the maximum measuring temperature (300 K) allowed by our experimental VSM setup and, consequently, the blocking temperature (T_B_) must be above 300 K. According to Kneller plot from Figure 5A, T_B_ should be 233 K for CFO73, and this value is the lowest T_B_ obtained among all samples. This discrepancy could be explained by the distribution of NPs size which causes deviation from linearity in the fit from Figure 5A at high temperatures. Moreover, one should keep in mind that the NPs under study are not isolated, but they form the walls of a nanoporous foam with open and closed pores and a model of non-interacting NPs is not completely true. On the other hand, the barrier height between the two possible magnetic states which describes the ZFC curves (along and opposite to applied magnetic field) depends on the applied magnetic field. In this case, it is possible to obtain a field-dependent maximum of the ZFC curve, which can provide the value of T_B_ as shown in Figure 6A for CFO73. The blocking temperature T_B_ (temperature where ZFC curve has maximum value) decreases with the increase in the applied magnetic field. For non-interacting NPs, the dependence of T_B_ vs. the applied magnetic field should be [33]:

(2) $$T_{B}\left(H\right)=T_{B}\left(0\right)\times {\left(1-\frac{H}{H_{K}}\right)}^{\alpha }$$

where T_B_(H) is the blocking temperature measured in applied field H whereas T_B_(0) is the blocking temperature measured in negligible magnetic field and H_K_ is the anisotropy magnetic field (the switching field in case of uniaxial anisotropy). The value H_K_ = 2 K/M_s_ for the switching magnetic field is the same for NPs with uniaxial magnetic anisotropy [30] and for NPs with cubic anisotropy when the magnetization is orientated on the (100) direction [31] which is the case of cobalt ferrite. The coefficient α from Equation (2) should be α = 2 for low magnetic fields [33] and α = 2/3 for high magnetic fields [34]. Taking into account that for a random distribution of easy magnetic axis for NPs with cubic anisotropy [30] H_c0_ = 0.64 K/M_s_ and using M_s_ given by the approach to saturation law at the lowest measurement temperature (10 K), it is clearly shown that the experimental points (T_B_, H) obey the dependence from Equation (2). From the fit presented in Figure 6B, one obtains the value T_B_ = 340(7) K extrapolated at an applied field H = 0 Oe and the exponent coefficient α = 1.9(1).

**Figure 7:**
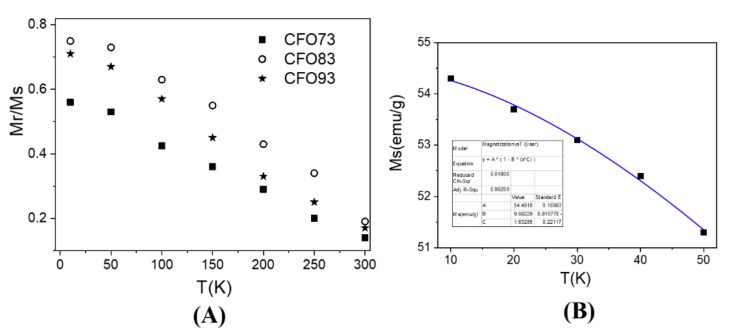
Mr/Ms for all samples (**A**) and thermal dependence of magnetization at saturation (Ms) for sample CFO73 (**B**).

Figure 7A depicts the evolution of the ratio between remanent magnetization (M_r_) and magnetization at saturation (M_s_) vs. temperature for all three samples and this ratio is denoted as R = M_r_/M_s_. The maximum R value was achieved at 10 K (lowest measuring temperature) and the lowest one at 300 K (highest measuring temperature). According to Stoner–Wohlfarth model [30], a value of R = 0.5 is obtained for an assembly of non-interacting single-magnetic-domain NPs with uniaxial symmetry assuming a random distribution of easy magnetic axes (in the magnetic frozen regime which corresponds experimentally to the lowest temperature data). For spherical NPs with cubic magnetocrystalline anisotropy, R should be 0.83 [31]. For our samples, the maximum R values measured at 10 K were 0.75 for CFO83, 0.71 for CFO93, and 0.56 for CFO73, and this clearly indicates a cubic magnetic anisotropy provided by the magnetocrystalline anisotropy of CoFe_2_O_4_ which exceeds the shape anisotropy effect. Among all the samples, the maximum value of R (0.75) was obtained for CFO83 at 10 K, and this value is close to the theoretical one (0.83). Of note is that the only expected interparticle interactions in our nanoparticulate system are of dipolar type which are antiferromagnetic (AF). The AF effect of interparticle interactions is to diminish the R value compared with the non-interacting case, and this experimental behavior was observed. As expected, due to the smallest crystallite size and, consequently, the highest spin distortion, the smallest value of R was found for CFO73. R values for all samples decrease not only with crystallite size but also with temperature increase due to spin fluctuations which become important at high temperatures (e.g., R should become zero at the blocking temperature). However, R = 0 was not reached either at 300 K, and this result demonstrates the non-complete magnetic relaxation even at this temperature, due to the two previously discussed factors (broad particle size distribution and interparticle interactions).

In Figure 7B is shown the low temperature dependence of M_s_ (obtained by approach to saturation law) for CFO73 vs. temperature. The curve can be well fitted with the equation

(3) $$M_{s}=M_{s}\left(0\right)\left(1-A\times T^{B}\right).$$

Here, M_s_(0) is the magnetization at saturation extrapolated at 0 K, and A and B are constants. The fit provides a value of 1.6(2) for exponent B, close to the theoretical value of 1.5 specific to the spin waves mechanism. Even though spin waves Bloch model was previously derived for an infinite crystal, further simulations were also performed for finite size nanometer clusters and follow the same law, but with slightly larger exponent parameter B [35]. The maximum value of magnetization at saturation (M_s_) measured at 10 K obtained from the law of approach to saturation was 54.3 emu/g for CFO73, 83.6 emu/g for CFO83, and 82.7 emu/g for CFO93. The same measurements at 300 K gives the values 37.9 emu/g for CFO73, 67.5 emu/g for CFO83, and 67.2 emu/g for CFO93. In the literature, it can be found that the M_s_ for bulk [36] CoFe_2_O_4_ is 80.8 emu/g when measured at ambient temperature, and 93.9 emu/g at 5 K. M_s_ for CoFe_2_O_4_ at 300 K decreases with 13.1 emu/g compared with M_s_ at 10 K. Consequently, the reason for this behavior is the variation with temperature of the electronic populations of hybrid orbitals. In comparison, for our samples (NP system, not bulk material) the decrease of M_s_ value when temperature varies from 10 to 300 K is 16.4 emu/g for CFO73, 16.1 emu/g for CFO83, and 15.5 emu/g for CFO93. The larger decrease of M_s_ for NPs compared with the bulk counterpart could be justified by thermal fluctuations of magnetic moment in a single energy minimum when temperature increases. At low temperatures, when spin orientation thermal fluctuations can be neglected, we can explain the difference between the M_s_ of the samples under study and M_s_ of bulk CoFe_2_O_4_ by the presence of the outer layer belonging to the NPs surface. The surface to volume ratio increases with the decrease of NP diameter. The outer layer of the NPs has a distorted spin structure and gives negligible contribution to M_s_. In the literature, it is given as an empirical formula [37] which correlates the M_s_ for NPs with the M_s_ for bulk samples of similar composition:

(4) $$M_{s}=M_{s}\left(bulk\right)\times \left(1-\frac{6g}{D}\right)$$

where *g* is the thickness of the shell (outer layer of the NP) and *D* is NP diameter. Using the mean diameter (*D*) obtained from TEM micrographs, M_s_ values measured at 10 K, and assuming M_s_(bulk) = 93.9 emu/g [36], we obtained shell thickness values of *g* = 0.55 nm for CFO73, *g* = 0.176 nm for CFO83, and *g* = 0.179 nm for CFO93. The shell thickness *g* corresponding to the “dead” magnetic layer is very small for CFO83 and CFO93, demonstrating that the NPs synthesized at these pH values are well formed, whereas for CFO73, the shell thickness value is several times higher, and the NPs are more defective. One explanation could be the larger BET surface area of CFO73 compared to that of CFO83 and CFO93. 

### 3.7. Mössbauer Measurements

**Figure 8:**
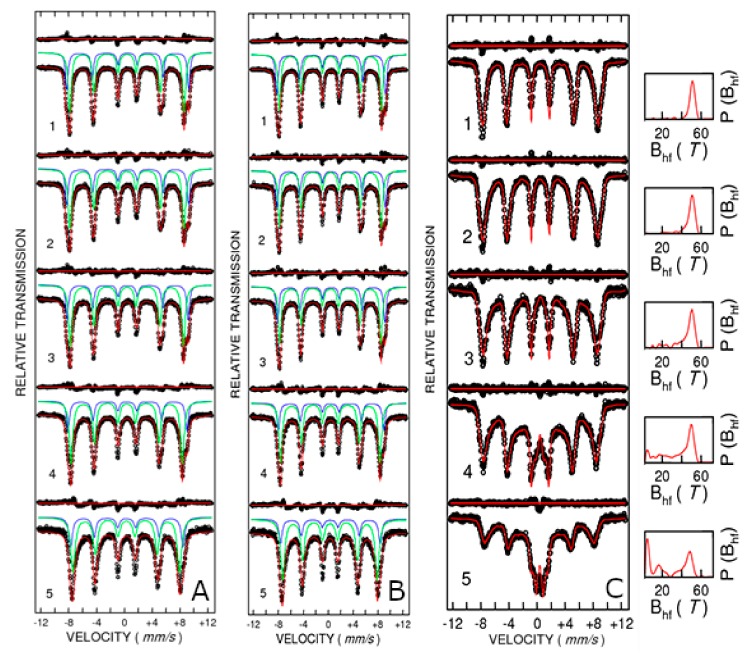
Mössbauer spectra at selected temperatures: 10 K (1), 50 K (2), 100 K (3), 200 K (4), 300 K (5) for samples CFO83 (**A**), CFO93 (**B**), and CFO73 (**C**).

^57^Fe Mössbauer spectroscopy was used to better describe the local electronic structure near Fe ion in the samples under study. It allows distinguishing among the iron contained in various phases or iron positions from the same phase but belonging to different crystallographic positions. A Mössbauer pattern contains sextets, doublets, or singlets, with each one corresponding to an iron non-equivalent position. A sextet corresponds to a magnetic environment to the iron nucleus whereas a doublet corresponds to non-zero electric field gradient to the nucleus. For these reasons, ^57^Fe Mössbauer spectroscopy is very useful to detect the composition of iron phases and Fe valence states, local distortion, cationic distribution, and magnetic behavior including relaxation of Fe magnetic moments via temperature evolution of the magnetic hyperfine field. Figure 8A,B shows the Mössbauer spectra measured at temperatures ranging from 10 to 300 K for samples CFO83 and CFO93, respectively. Cobalt ferrite (Co^2+^Fe^3+^_2_O^2−^_4_) in the ideal case is an inverse spinel with Fe^3+^ equally distributed among tetrahedral and octahedral positions. In the real case, the distribution of Fe^3+^ either on tetrahedral or octahedral positions depends on the thermal history of the sample and preparation route and, consequently, the spinel is only partially inverse. The Mössbauer spectra were properly fitted at all measuring temperatures with two spectral components (sextets) which belong to the tetrahedral and octahedral Fe^3+^ positions. The isomer shift values are within the range of values 0.4–0.5 mm/s at the lowest measuring temperature and 0.3–0.4 mm/s at 300 K (relative to metallic iron) which are characteristic of the Fe^3+^ valence state. The modification of the isomer values to shift toward positive values at low temperatures is due to the well-known second- order Doppler shift. No spectral components with higher isomer shift corresponding to Fe^2+^ can be detected in the Mössbauer pattern. Consequently, only Fe^3+^ oxidation state occurs in the sample. Taking into consideration the stoichiometric formula CoFe_2_O_4_ derived from EDAX measurements due to electric neutrality per formula unit, the oxidation state of cobalt should be Co^2+^, as usually encountered in cobalt ferrites. 

The quadrupole splitting values are small, demonstrating only minor local distortion. The outer sextet with higher hyperfine magnetic field of about 54.5 T at the lowest measuring temperature belongs to the tetrahedral Fe^3+^ position, whereas the sextet of about 51.1 T at the same temperature corresponds to the octahedral Fe^3+^ position for the CFO83 sample. At 300 K, these hyperfine field values are lower, 50.1 T for tetrahedral Fe^3+^ and 47.5 T for octahedral Fe^3+^. These values are close for the CFO93 sample: H = 54.1 T for tetrahedral Fe^3+^ and H = 51 T for octahedral Fe^3+^ at 10 K, and H = 49.4 T for tetrahedral Fe^3+^ and H = 47 T for octahedral Fe^3+^ at 300 K. Mössbauer spectra showing two well-demonstrated sextets with similar hyperfine field values as obtained in the present work, corresponding to Fe^3+^ located in octahedral and tetrahedral positions, were previously reported in literature as follows. Grigorova et al [36]. found H = 54.3 T for tetrahedral Fe^3+^ and H = 51.3 T for octahedral Fe^3+^ at 80 K for cobalt ferrite NPs of about 20 nm obtained by cobalt–iron hydroxide carbonate co-precipitation route. Ngo et al. [38] found H = 53.6 T for tetrahedral Fe^3+^ and H = 51.2 T for octahedral Fe^3+^ at 4.2 K for cobalt ferrite NPs of 6.2 nm obtained by normal micelles method using sodium dodecyl sulfate Na(DS). Chandra et al. [39] found H = 50.0 T for tetrahedral Fe^3+^ and H = 47.7 T for octahedral Fe^3+^ at 300 K for cobalt ferrite NPs of 10 nm obtained by sol–gel technique using citric acid. ^57^Fe Mössbauer spectroscopy shows the absence of α-Fe_2_O_3_ (hematite), even in small amount or amorphous form, which would be difficult to detect by XRD. Moreover, ^57^Fe Mössbauer spectroscopy rules out the occurrence of other iron-containing oxides like FeO (wüstite) or Fe_3_O_4_ (magnetite), according to the isomer shift and hyperfine magnetic field values, which demonstrate the cobalt ferrite purity of our samples. The relative areas corresponding to the two sextets belonging to octahedral and tetrahedral Fe^3+^ positions allow us to extract the Fe^3+^ distribution among the tetrahedral and octahedral positions, the most precise results being obtained in the magnetic frozen regime. At the lowest measuring temperature for CFO83 sample, there was a resulting 64% Fe^3+^ on octahedral sites and 36% Fe^3+^ on tetrahedral sites, whereas for CFO93 sample, the distribution is 61% Fe^3+^ on octahedral sites and 39% Fe^3+^ on tetrahedral sites. There is no evidence of relaxation processes with increasing temperature in the Mössbauer pattern corresponding to CFO83 and CFO93 (Figure 8A,B, respectively). On the other hand, Mössbauer spectra of CFO73 measured at various temperatures (Figure 8C) exhibit a relaxation process characterized by a sextet with broad lines overlapping with a central doublet, and by the distribution of the hyperfine magnetic fields which become wider with the rise in temperature and shift to smaller average hyperfine fields. However, the Mössbauer pattern of CFO73 measured at low temperatures, where the relaxation effect is negligible, resembles that of CFO83 and CFO93, demonstrating the formation of cobalt ferrite without the existence of any parasite phases. The hyperfine field and occupancy values for CFO73 corresponding to tetrahedral Fe^3+^ and octahedral Fe^3+^ measured at 10 K are 53.2 T and 40%, and 50.2 T and 60%, respectively, close to the values obtained for CFO83 and CFO93. Expectedly, the values obtained for the hyperfine field increase with the crystallite size for a given temperature (H(CFO83) > H(CFO93) > H (CFO73)) for both the tetrahedral and octahedral Fe^3+^ sites due to more defected structure and increased relaxation phenomena occurring for smaller crystallites.

The occupancy ratios between tetrahedral and octahedral Fe^3+^ obtained from Mössbauer fit allow us to find the chemical formulas (spinel type AB_2_O_4_ where A—tetrahedral and B—octahedral positions): (i) (Co_0.28_ Fe_0.72_) (Co_0.72_ Fe_1.28_) O_4_ for CFO83, (ii) (Co_0.22_ Fe_0.78_) (Co_0.78_ Fe_1.22_) O_4_ for CFO93, and (iii) (Co_0.2_ Fe_0.8_) (Co_0.8_ Fe_1.2_) O_4_ for CFO73. 

In order to find out these results, we assumed that (i) there is one atom in total on the tetrahedral A position and two atoms on the octahedral B position; (ii) there are 1 Co and 2 Fe atoms per formula unit according to stoichiometry from EDAX measurements; (iii) the ratio between the number of Fe^3+^ atoms from octahedral and tetrahedral positions is derived from the relative area of the two spectral components from Mössbauer pattern; and (iv) Fe is present in the samples only in trivalent state (Fe^3+^) according to Mössbauer data. Taking into consideration that iron is trivalent (Fe^3+^) and gives a spin contribution to the magnetic moment of 5µ_B_ and cobalt is divalent (Co^2+^) and provides 3µ_B_ and recalling that in the ferrimagnetic spinel structure, the magnetic moment of octahedral B positions is antiparallel to the magnetic moment of tetrahedral A positions, we obtain the following values for the magnetic moment per formula unit (f.u.) on the basis of the chemical formulas derived just above: (i) 4.2 µ_B_/f.u. for CFO83, (ii) 3.88 µ_B_/f.u. for CFO93, and (iii) 3.8 µ_B_/f.u. for CFO73. Next, the theoretical values of magnetization at saturation (M_s_) estimated at low temperatures (from Mössbauer data) have been obtained using these values of magnetic moment per f.u. We obtained 98 emu/g for CFO83, 92.3 emu/g for CFO93, and 90.5 emu/g for CFO73. These values are close to that reported in literature for bulk CoFe_2_O_4_ measured at low temperatures, M_s_(bulk) = 93.9 emu/g [36]. M_s_ depends on Fe distribution among tetrahedral and octahedral sites and is possible to exceed for a specific cationic configuration (CFO83 nanoparticulate system) the value corresponding to bulk ferrite. One should recall that the maximum experimental values of M_s_ measured at 10 K obtained from approach to saturation law were 54.3 emu/g for CFO73, 83.6 emu/g for CFO83, and 82.7 emu/g for CFO93. Using the experimental values of M_s_ and the theoretical ones derived from Mössbauer results and Equation (4), one can estimate the magnetic “dead” magnetic layer thickness of 0.52 nm for CFO73, 0.23 nm for CFO83 and 0.16 nm for CFO93. Also in this case, the maximum thickness of the NPs in the outer nonmagnetic layer was found for CFO73, which seems to have the most defective structure among all the analyzed samples, which is plausible taking into consideration that CFO73 has also maximum BET surface. 

**Figure 9:**
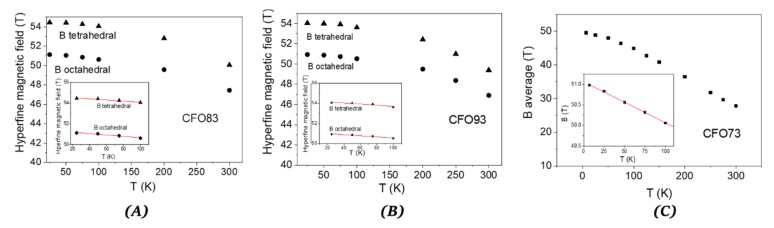
Thermal dependence of the hyperfine magnetic field for CFO83 (**A**), CFO93 (**B**), and CFO73 (**C**).

Figure 9A,B, show the dependence on temperature of the hyperfine magnetic field for tetrahedral and octahedral Fe^3+^ for samples CFO83 and CFO93, respectively, whereas for sample CFO73, the variation with temperature (Figure 9C) of the field is given by the peak from the magnetic field distribution. The variation of the hyperfine magnetic field at low temperatures is given by the “collective excitation regime” [40] which is characterized by lowering the average field value with temperature increase due to small oscillations of the magnetic moment around the easy magnetization axis. This is not yet the superparamagnetic true regime when the orientation of the magnetic moment of the NP can change to the opposite direction. From the Mössbauer spectroscopy point of view, the “collective excitation regime” is described by the decrease of the magnetic hyperfine field with temperature increase according to the formula:

(5) $$B\left(T\right)=B_{0}\left(\;1\;–\;\frac{k_{B}T}{\propto KV}\right)$$

where B(T) is the hyperfine magnetic field at absolute temperature T, B_0_ is its extrapolated value at 0 K, k_B_ is the Boltzmann constant, K is the magnetic anisotropy constant and V is the NP volume. The coefficient α depends on the type of magnetic anisotropy: α = 2 for uniaxial magnetic anisotropy [40] and α = 1.54 for cubic magnetic anisotropy [41], which is our case. The dimension of the magnetic core of the NPs can be calculated using the NPs average size given by TEM micrographs and subtracting the thickness of the dead magnetic layer (DL) according to D_magnetic_ = D_TEM_ − 2DL (see also Table 2). In the inset of Figure 9A–C are represented the linear fits of B vs. T at low temperatures corresponding to “collective excitation regime” described by Equation (5). From the slope of the fit and using the magnetic volumes obtained by the method described above, we can estimate the magnetic anisotropy constant (K) of the NPs: 2.93∙10^5^, 2.04∙10^5^, and 2.64∙10^5^ J/m^3^ for CFO73, CFO83, and CFO93, respectively. These results also support the information derived from coercive field dependence on temperature, which shows that the NPs with the lowest size contained in CFO73 have also the highest anisotropy constant among all samples. Simple hydrothermal synthesis yields non-mesoporous cobalt ferrite NPs of 34 nm with cubic/spherical shape and M_s_ = 56.8 emu/g (300 K), and their non-porous nature makes them unsuitable as drug vehicles [42]. Using the co-precipitation–annealing (at 1000 °C) method [43] NPs of cobalt ferrite were formed with elongated morphologies (80–160 nm length and 43 nm width) exhibiting Ms = 43.7 emu/g, lower than the result afforded by the method reported here. Monodisperse mesoporous cobalt ferrite NPs obtained by a multi-step route involving a ligand-exchange method have been reported recently, but these exhibit inferior magnetic properties at ambient temperature (55.1 emu/g for CoFe_2_O_4_ RNA, compared to our value of 68 emu/g) [44]. All these previous results given in literature confirm the advantages of the facile hydrothermal surfactant-assisted method reported in the present work, suitable for obtaining mesoporous structures with high magnetization for saturation at ambient temperature. 

## 4. Conclusions

A cheap and facile method to produce mesoporous cobalt ferrite with high BET surface area and significant magnetization at saturation at ambient temperature has been proposed. This technique is a surface-assisted hydrothermal method using Pluronic non-ionic surfactant. The mesoporous structures are built from nanometer-sized crystallites. The key factor affecting the crystallite dimensions as well the phase purity was the pH value. A parasite hematite phase begins to form at pH values lower than 7. Depending on initial pH conditions, these mesostructures are built from crystallites of 7.8, 9.6, and 9.0 nm. A high BET surface area of about 151 m^2^/g was obtained for the sample prepared at pH 7.3. There is a significant decrease in the magnetization at saturation from 10 to 300 K (e.g., from 83.6 to 68 emu/g for samples obtained at pH 8.3) due to both changing of the population of electron orbitals and thermal fluctuations of the magnetic moment. Non-zero magnetic coercive fields of a few hundred Oe were noticed in the hysteresis curves at 300 K, demonstrating that a small portion of the NPs are not superparamagnetically relaxed even at this temperature, due to the inherent distribution of crystal size and dipolar interactions among the magnetic moments of the crystallites. ^57^Fe Mössbauer spectroscopy measurements demonstrated the cobalt ferrite phase purity of our samples and allowed Fe occupancy of the tetrahedral and octahedral positions of the cobalt ferrite spinel. In this way, we calculated the theoretical magnetization at saturation, and by comparing it with the experimental values, we obtained the thickness of the “dead” magnetic outer layer of the NPs. Using the hyperfine magnetic field dependence on temperature, we estimated the magnetic anisotropy constant (K) of the NPs as 2.93∙10^5^, 2.04∙10^5^, and 2.64∙10^5^ J/m^3^ for the samples obtained at pH values 7.3, 8.3, and 9.3, respectively. Taking into consideration the high BET surface area and K values and also the significant magnetization at saturation at ambient temperature, possible applications of these materials as recyclable anti-polluting agents, magnetically separable catalysts, and targeted drug delivery vehicles could be of high interest. 

## Figures and Tables

**Table 1:** Morphological characteristics of as-synthesized cobalt ferrite CoFe_2_O_4._

CoFe_2_O_4_—pH of Synthesis	BET Surface Area (m^2^/g)	Pore Volume (cm^3^/g)	Pore Diameter (nm)	Phase(s) Present (p-XRD)	Obs.
6.4	56	0.296	15.50	Fe_2_O_3_ (minor), CoFe_2_O_4_ (main)	Fe_2_O_3_—hematite parasite phase
7.3 (CFO73)	151	0.312	5.42	CoFe_2_O_4_	
8.3 (CFO83)	101	0.340	11.46	CoFe_2_O_4_	
9.3 (CFO93)	88	0.310	11.42	CoFe_2_O_4_	

**Table 2:** Parameters extracted from Rietveld refinements of XRD data and TEM and magnetic size of the nanoparticles (NPs).

SAMPLE	Lattice Parameter (nm)	rms Microstrain (%)	XRD Crystal Size (nm)	TEM Average Size (nm)	Magnetic Size (nm)	Fit Reliability Parameters (R_B_, R_wp_, GOF)
CFO73	0.8369 (7)	0.001 (2)	5.4 (3)	7.8	6.7	R_wp_ = 1.05% R_B_ = 0.71% GOF = 1.09
CFO83	0.8368 (5)	0.0014 (9)	8.3 (5)	9.6	9.1	R_wp_ = 0.82% R_B_ = 0.55% GOF = 1.05
CFO93	0.8371 (5)	0.002 (1)	7.8 (4)	9.0	8.7	R_wp_ = 0.90% R_B_ = 0.57% GOF = 1.08

## Supplementary Materials

The following are available online at https://www.mdpi.com/2079-4991/10/3/476/s1, Figure S1: XRD spectra of poorly crystallized CoFe_2_O_4_ samples obtained at pH 10.3 and 11.3; Figure S2: TGA-DSC coupled curves showing surfactant (P123 Pluronic) decomposition; Figure S3: FTIR spectra of cobalt ferrite samples CFO73, CFO83 and CFO93 (from bottom to top).
