# Supplementary material for: Mesoporous Cobalt Ferrite Nanosystems Obtained by Surfactant-Assisted Hydrothermal Method: Tuning Morpho-structural and Magnetic Properties via pH-Variation
Source: Nanomaterials (Basel). 2020 Mar 6;10(3):476. doi: 10.3390/nano10030476 (PMC7153623; doi:10.3390/nano10030476)
Supplement: Supplementary file 1 [file nanomaterials-10-00476-s001.pdf]

Mesoporous Cobalt Ferrite Nanosystems Obtained by Surfactant-Assisted Hydrothermal Method: Tuning Morpho-structural and Magnetic Properties via pH-Variation

Petru Palade [1], Cezar Comanescu [1,*], Andrei Kuncser [1], Daniela Berger [2], Cristian Matei [2], Nicusor Iacob [1] and Victor Kuncser [1]

[1] National Institute of Materials Physics, Atomistilor 405A, 077125 Magurele, Romania; palade@infim.ro (P.P.); akuncser@yahoo.com (A.K.); nicusor.iacob@infim.ro (N.I.); kuncser@infim.ro (V.K.)

[2] Polytechnic University of Bucharest, Faculty of Applied Chemistry and Materials Science 1–7 Polizu St, Bucharest 011061, Romania; danaberger01@yahoo.com (D.B.); cristi_matei@yahoo.com (C.M.)

* Correspondence: cezar.comanescu@infim.ro

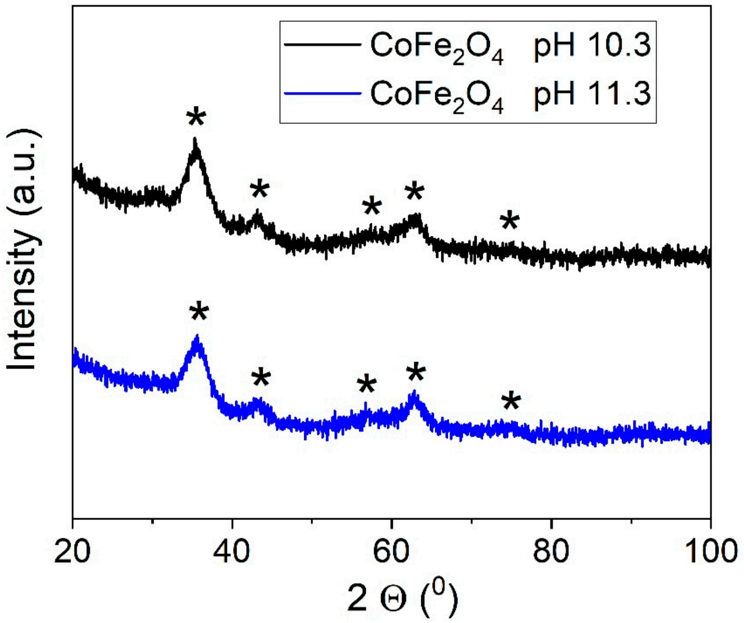

Figure S1. XRD spectra of poorly crystallized $CoFe_2O_4$ samples obtained at pH 10.3 and 11.3.

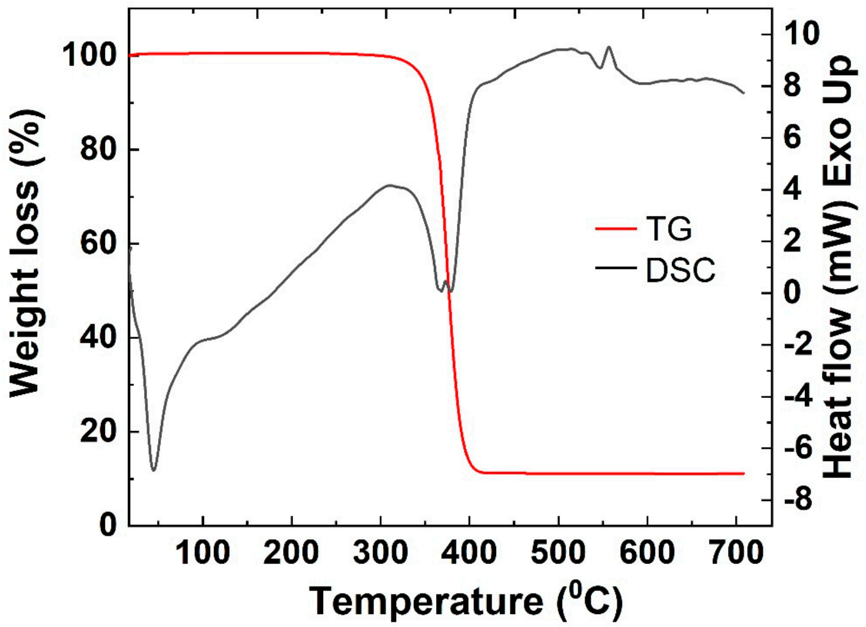

Figure S2. TGA-DSC coupled curves showing surfactant (P123 Pluronic) decomposition.

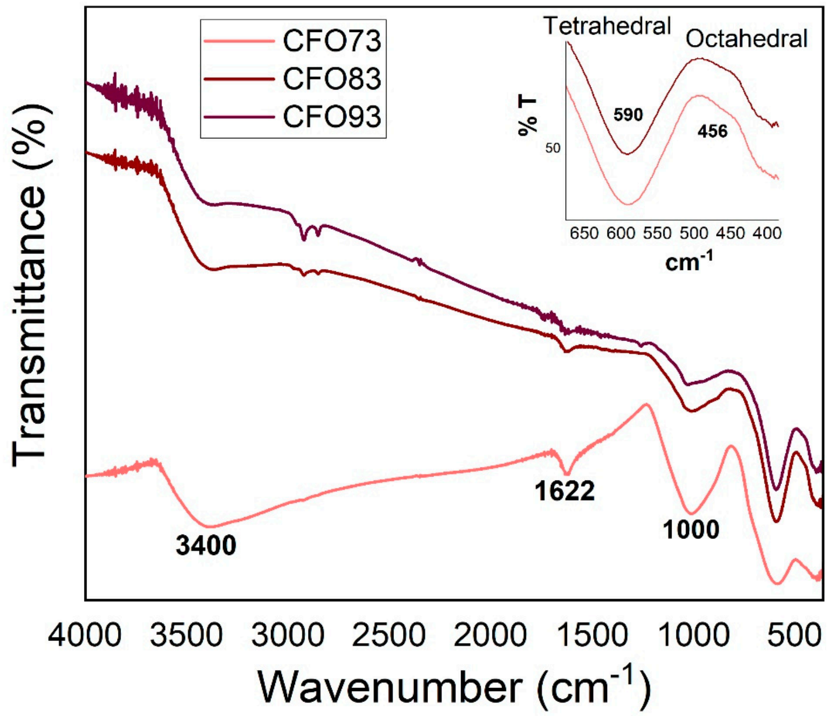

Figure S3. FTIR spectra of cobalt ferrite samples CFO73, CFO83 and CFO93 (from bottom to top).
